# Supplementary material for: Predictive Value of Precision-Cut Lung Slices for the Susceptibility of Three Animal Species for SARS-CoV-2 and Validation in a Refined Hamster Model
Source: Pathogens. 2021 Jun 30;10(7):824. doi: 10.3390/pathogens10070824 (PMC8308566; doi:10.3390/pathogens10070824)
Supplement: Supplementary file 1 [file pathogens-10-00824-s001.zip › pathogens-1234020-suppl-final.pdf]

Supplementary materials and methods

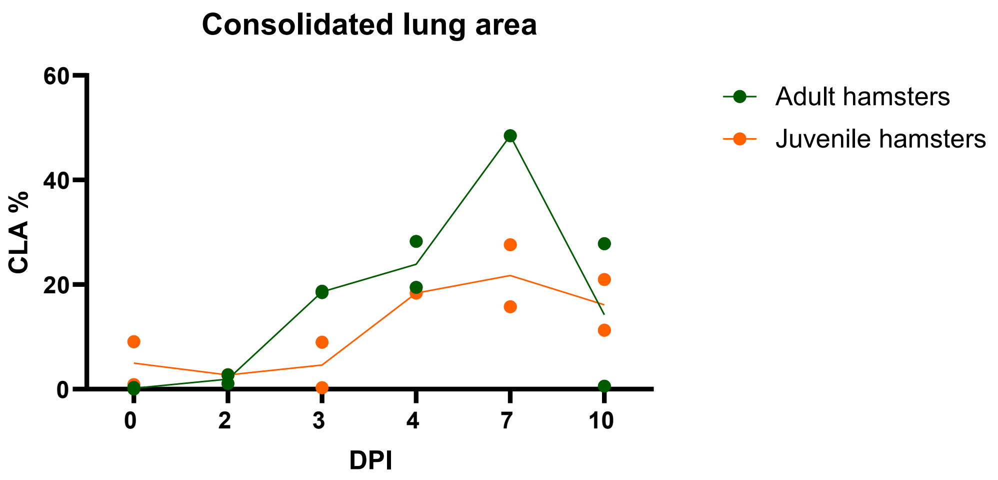

Supplementary Figure S1. Consolidated lung area (CLA), calculated based on photographs that were taken during necropsy. Dorsal and ventral images of lungs were taken and analyzed with Image Pro Premier 64-bit software, which calculates the percentage of discolored tissue surface relative to the whole organ surface. On each day post infection (DPI), N=2 animals per group were analyzed, except on DPI 4 and 7, where only one lung from a juvenile and an adult hamsters was analyzed respectively. Symbols show individual values and lines represent the average. DPI – days post infection.

Supplementary Table S1 Numerical values of the total and subgenomic viral RNA copy numbers [log10/swab or gram tissue] and virus titers [log10 TCID 50/swab or gram tissue] grouped by sample type (swabs, conchae, lungs and trachea) and days post infection (DPI). Averages and standard deviations are shown.

	**Adult hamsters**		**Juvenile hamsters**	
Swabs: total E-gene PCR				
	Average log10 RNA copy number/swab	SD	Average log10 RNA copy number/swab	SD
DPI 2-7	7.57	0.25	7.43	0.43
DPI 10	5.61	0.53	4.59	0.42
DPI 21	2.07	1.21	1.00	0.00
Swabs: sub-genomic PCR				
	Average log10 RNA copy number/swab	SD	Average log10 RNA copy number/swab	SD
DPI 2-7	5.57	0.29	5.35	0.43
DPI 10	3.11	0.71	2.71	0.23
DPI 21	not tested		not tested	
Conchae: total E-gene PCR				
	Average log10 RNA copy number/gram tissue	SD	Average log10 RNA copy number/gram tissue	SD
DPI 2-7	11.61	0.25	10.71	0.50
DPI 10	8.60	0.13	7.88	0.27
DPI 21	5.33	2.77	6.77	1.29
Conchae: sub-genomic PCR				
	Average log10 RNA copy number/gram tissue	SD	Average log10 RNA copy number/gram tissue	SD
DPI 2-7	10.36	0.28	9.62	0.53
DPI 10	3.31	3.27	not detected	
DPI 21	not detected		not detected	
Conchae: virus titer				
	Average log10 TCID50/gram tissue	SD	Average log10 TCID50/gram tissue	SD
DPI 2-4	6.71	0.42	6.49	0.51
DPI 7 and 10	not detected		not detected	
DPI 21	not tested		not tested	
Lungs: total E-gene PCR				
	Average log10 RNA copy number/gram tissue	SD	Average log10 RNA copy number/gram tissue	SD
DPI 2-4	11.11	0.29	11.49	0.36
DPI 7 and 10	7.59	0.32	7.78	0.30
DPI 21	6.55	0.25	6.62	0.20
Lungs: sub-genomic PCR				
	Average log10 RNA copy number/gram tissue	SD	Average log10 RNA copy number/gram tissue	SD
DPI 2-4	9.59	0.31	9.95	0.34
DPI 7 and 10	not detected		not detected	
DPI 21	not detected		not detected	

	Adult hamsters		Juvenile hamsters	
Lungs: virus titer				
	Average log10 TCID50/gram tissue	SD	Average log10 TCID50/gram tissue	SD
DPI 2-4	6.15	0.42	6.05	0.55
DPI 7 and 10	not detected		not detected	
DPI 21	not tested		not tested	
Trachea: total E-gene PCR				
	Average log10 RNA copy number/gram tissue	SD	Average log10 RNA copy number/gram tissue	SD
DPI 2-4	8.95	0.48	8.57	0.93
DPI 7 and 10	6.52	0.68	5.29	0.76
DPI 21	3.32	1.96	3.98	1.97
Trachea: sub-genomic PCR				
	Average log10 RNA copy number/gram tissue	SD	Average log10 RNA copy number/gram tissue	SD
DPI 2-4	7.65	0.43	7.37	0.88
DPI 7 and 10	not detected		not detected	
DPI 21	not detected		not detected	
Trachea: virus titer				
	Average log10 TCID50/gram tissue	SD	Average log10 TCID50/gram tissue	SD
DPI 2-4	1.52	1.27	not detected	
DPI 7 and 10	not detected		not detected	
DPI 21	not tested		not tested	

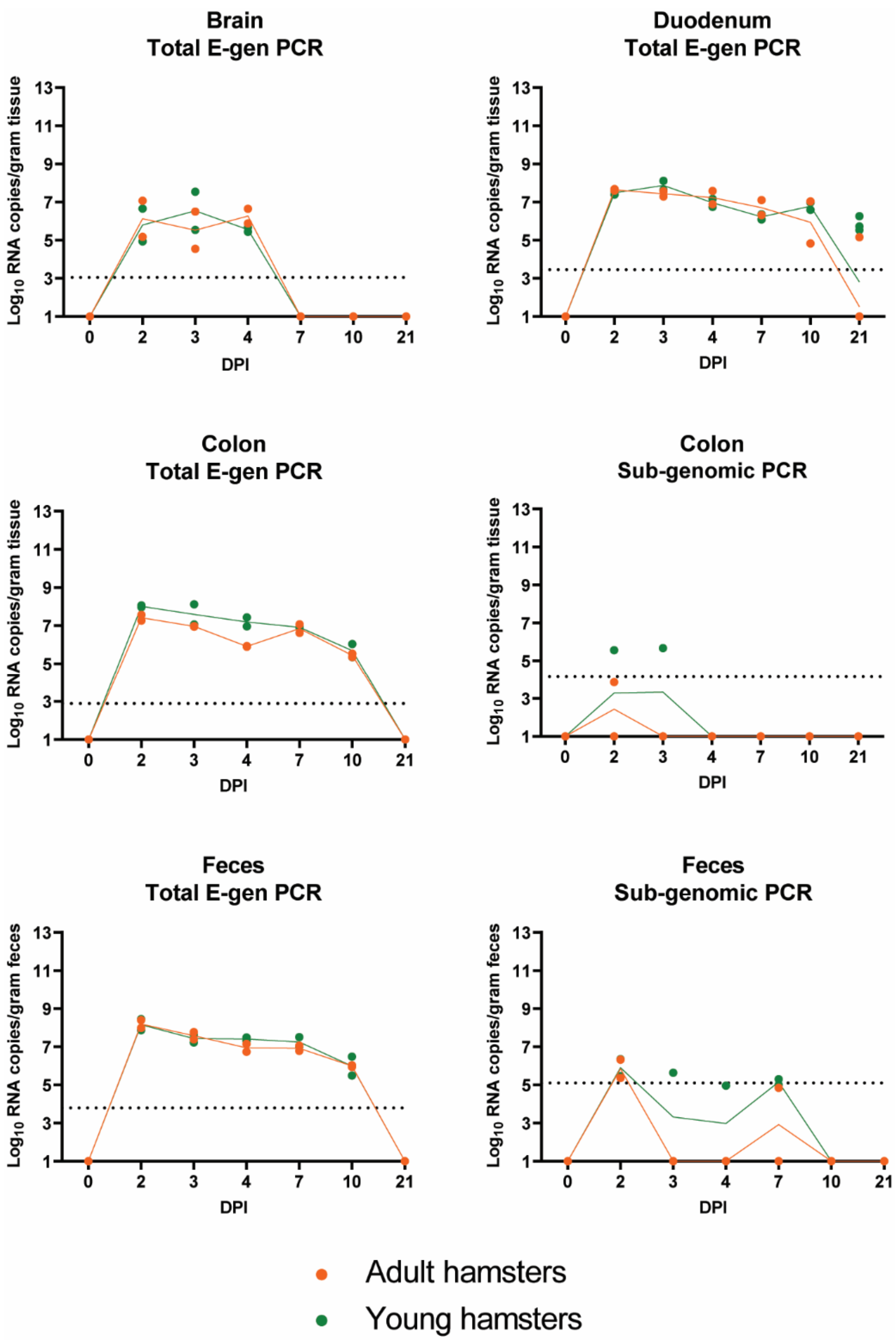

Supplementary Figure S2. SARS-CoV-2 viral loads in non-respiratory tissues. RNA quantities were determined by RT-qPCR and are expressed as log10 RNA copies per gram of tissue. Each symbol represents a value of an individual animal and the lines show means. On DPI 0 - 10, N=2 animals per group were analyzed, and on DPI21 – N=8 per group. Horizontal dotted lines show limit of detection of each assay. Samples displayed with a value of "1" had undetectable virus or RNA content. DPI – days post inoculation.
